# Teriparatide improves early callus formation in distal radial fractures: Analysis of a subgroup of patients within a randomized trial

**DOI:** 10.3109/17453671003761946

**Published:** 2010-04-06

**Authors:** Per Aspenberg, Torsten Johansson

**Affiliations:** Division of Orthopaedics, Department of Clinical and Experimental Medicine, Faculty of Health Sciences, Linköping UniversitySweden

## Abstract

**Background** Teriparatide (parathyreoid hormone; PTH 1-34) increases skeletal mass in humans and improves fracture healing in animals. A recent randomized multicenter trial of nonoperated distal radial fractures showed a moderate shortening of the time to restoration of cortical continuity by treatment with 20 μg (low-dose) teriparatide per day, but not with 40 μg (high-dose). As radiographic cortical continuity appears late in the healing process, perhaps too late for clinical relevance, we studied the qualitative appearance of the callus 5 weeks after fracture.

**Methods** One third of the patients of the international trial were treated at Linköping University Hospital. The multicenter trial did not evaluate early callus formation. We therefore made a blinded qualitative scoring of the callus at 5 weeks in our 27 patients. Callus formation was arbitrarily classified as rich, intermediate, or poor.

**Results** 9 patients were classified as rich (none had received placebo, 3 low-dose teriparatide, and 6 high-dose teriparatide). 9 patients were classified as intermediate (1 had received placebo, 5 low-dose, and 3 high-dose). 9 patients were classified as poor (7 had received placebo, 1 low-dose, and 1 high-dose) (p < 0.001).

**Interpretation** This is a post hoc subgroup analysis of an outcome variable, which was not in the official protocol. The results must therefore be interpreted with caution. However, in combination with the results of the larger trial, the data suggest that radiographic quality at an early time point might be a sensitive variable, perhaps better than time to cortical continuity. Moreover, teriparatide appeared to improve early callus formation in distal radial fractures.

## Introduction

Based on animal and clinical data, there have been high expectations that intermittent treatment with parathyroid hormone (PTH) would be efficient for acceleration of fracture healing (Chalidis et al. 2007, Skripitz and Aspenberg 2004). Low intermittent doses, given over more than a year, increase bone density and reduce fracture risk in humans. However, the doses used in animals for fracture healing studies have been higher than what can be given to humans. On the other hand, animal data suggest that fracture healing responds more strongly to PTH than does bone density (Skripitz et al. 2000).

In a recent randomized trial, teriparatide (PTH 1-34) was tested in nonoperatively treated distal radial fractures (Aspenberg 2009). The study compared placebo, teriparatide 20 μg, and teriparatide 40 μg, given for 8 weeks. The patients were radiographed regularly, and the primary endpoint was time to cortical continuity in 3 of the 4 visible contours of the volar and radial projections. The main hypothesis was that 40 μg would shorten the time to continuity, and the lower dose was only to be tested if that were the case. No effect of 40 μg teriparatide was found. Even so, all 3 groups were still compared and found to show significant differences; 20 μg teriparatide appeared to have shortened the time to cortical continuity by 2 weeks (p = 0.006).

During the planning of this study, it was debated whether we should use estimations of a variable at a single time point or whether time to a certain event should be used. This issue has been discussed recently at conferences designed to work out guidelines for such clinical trials, but no consensus has been reached (Goldhahn et al. 2008). It was decided to do the trial as in the study described above. However, in Linköping we were in any case eager to study a rather early single time point. This was not because we were convinced that this would have greater statistical strength, but rather that it would have more clinical relevance.

As it turned out, the local sub-study in Linköping gave a clear-cut positive result, and as it could not be included in the report of the large trial, it is reported here separately.

## Patients and methods

27 women were included at the Linköping study site. All were over 50 years of age (median 64, maximum 80), with minimal or no other health problems and a dorsally dislocated distal radial fracture that had required closed reduction but was deemed appropriate for nonoperative treatment. No strict radiographic inclusion criteria were defined, as this was dependent on clinical traditions of the different study sites, and the choice of nonoperative treatment should be based on the clinical situation rather than on radiographs. In Linköping, included patients had a pre-reduction dorsal angle roughly between 5 and 30 degrees and discontinuities of the radial joint surface of less than 2 mm. Main exclusion criteria were: concomitant injury, previous wrist fracture, malignant neoplasm during the previous 5 years, liver disease, high calcium levels, joint disease, or a disease affecting bone metabolism (Aspenberg et al. 2009). The protocol for the multicenter study was followed rigorously. No patient was lost to follow-up. A site visit by the Swedish Medial Agency gave a good report.

Our evaluation was done after the last patient had been included, during the postoperative year, which had to pass before unblinding. After looking at series of digital radiographs for a number of patients, we decided only to use the pre-reduction images and those at the 5-week follow-up. We asked ourselves: “Assuming that PTH has a positive effect on fracture healing, do we think this patient received PTH?” and answered yes or no. After we had categorized all 27 patients, we went back and tried to describe the criteria that we had used.

This description was as follows. We (the authors) tried to get a general impression of the amount and density of callus, relative to what should be expected for that fracture type. We started with the lateral projection. In most cases, there is a sharp angle in the dorsal cortical contour at the fracture. At this angle there is usually an external callus. Most of our interest was focused on this callus. If absent, the case was rated as “placebo”. If there was a large or dense callus, it was rated as “PTH”. In cases of uncertainty, we looked for callus in other areas, i.e. at the volar cortex or at the sides in the anteroposterior projection. We paid no attention to the fracture line or to cortical continuity. We sometimes discussed the appearance of the cancellous bone, but generally concluded that this should not be included in the judgement. Fractures with little dislocation were expected to produce smaller external callus. In these cases, we looked more at callus density than at its size. We looked at the radiographs together and made consensus decisions.

We repeated the rating procedure 3 days later without seeing the previous results. 2 patients differed in rating between these time points. This latter rating was regarded as the definitive one and was reported to the sponsor who, at this time, did not inform us about whether or not the rating had been successful.

After the official outcome of the entire multicenter study was clear, but while we were still blinded, we were asked to do the same rating for all patients from all sites. This was not possible, however, because many patients at the other sites had had their 5-week radiographs taken before the plaster was taken off, and the images could not be evaluated. We therefore rated only the patients from Linköping again, almost a year after the first rating. This time, we rated the callus as rich, intermediate, or poor.

## Results

### Rating

In the first rating, we were able to judge from the 5-week radiographs which patients had received PTH (p = 0.03; Table 1).

**Table 1.** First rating

Guess	Treatment	
	Placebo	PTH
Placebo	6	5
PTH	2	14

The second rating correlated with treatment (Spearman's coefficient r_s_ 0.662 (95% CI: 0.376–0.832) p < 0.001). 9 cases were classified as having rich callus. All of them had been treated with PTH. In contrast, none of the placebo patients were classified as having rich callus (Table 2).

**Table 2.** Second rating

Rating	Treatment		
	Placebo	20 μg PTH	40 μg PTH
Poor	7	1	1
Intermediate	1	5	3
Rich	0	3	6

To estimate reproducibility, the second rating was later dichotomized to “placebo” or “PTH” (the latter comprising intermediate and rich callus). The first and second ratings then showed agreement in 23 of the 27 patients.

### Comparison with time to cortical continuity.

The second rating was compared with the data for time to cortical continuity from the multicenter study (3 out of 4 cortices). There was a correlation between time to continuity in Linköping and teriparatide dose (0, 20, or 40 μg) with a Spearman's coefficient r_s_ of 0.43 (95% CI: 0.063–0.70 p = 0.024). Dividing the times in tertiles gave r_s_ = 0.413 (95% CI: 0.039–0.686) p = 0.032). Remarkably, there was no strong correlation between the two radiological methods: r_s_ = 0.410 (95% CI: 0.036–0.684) p = 0.034.

## Discussion

How much can an analysis of a subgroup outside the protocol be trusted? We believe that the 5-week classification of patients was trustworthy. We regarded this variable as an unofficial primary endpoint. We had decided to look at this variable from the start, because we regarded it as being more relevant from our point of view, as we were most curious to see whether there is a biological response to PTH in human fracture healing (based on our previous experience with animals). The sponsor, however, was forced to concentrate on healing time because it was regarded as being more closely related to clinical application by the American Food and Drug Administration.

In contrast to the the main study, our sub-study showed a tendency to a more prominent effect in the high-dose PTH group. As the main study examined cortical bridging, its results might be sensitive to increased remodeling during the later stages of healing, so that the higher PTH dose may have made the forming cortex less visible on radiographs. This would be less of a problem during early healing.

Our study was mainly concerned with the effects of PTH on human fracture repair. It was not our primary goal to improve the clinical outcome. In the main study, few differences were seen between the treatment groups concerning grip strength and function, using PRWE (patient-related wrist evaluation score). There were no differences regarding palmar and radial displacement angles, but both PTH groups showed less radial shortening than the placebo group (p = 0.01). However, the combination of these results indicates that the clinical value of PTH treatment on distal radial fractures, if there is any, is limited.

Several drugs are currently being tested for improvement of fracture repair, but there is still no consensus regarding which outcome variables should be used. Radiography has (at most) a moderate relation to clinical outcome; also, if radiography has been chosen, it is unclear whether one should take measurements at a single time point or measure the time to the occurrence of some criterion for healing. Although teriparatide had an influence on both the appearance of the callus at 5 weeks and the time to cortical continuity, the correlation between callus rating at 5 weeks and healing times was not impressive. This may reflect the fact that the two evaluation methods measure different aspects of healing. A strong early callus response—in this case at 5 weeks—is possibly the more important of the two.

We are reporting our subgroup results to help somewhat in the ongoing discussions both about the use of PTH and about radiographic outcome criteria. We are aware that the correct approach would have been to first confirm our findings with a repeat study, with a protocol for qualitative assessment at 5 weeks. Still, our data add to the picture of the possible usefulness of PTH for fracture repair in general.
